# Two-dimensional semiconductor transistors and integrated circuits for advanced technology nodes

**DOI:** 10.1093/nsr/nwae001

**Published:** 2024-01-04

**Authors:** Weisheng Li, Haoliang Shen, Hao Qiu, Yi Shi, Xinran Wang

**Affiliations:** National Laboratory of Solid-State Microstructures, School of Electronic Science and Engineering and Collaborative Innovation Center of Advanced Microstructures, Nanjing University, China; Suzhou Laboratory, China; The Interdisciplinary Research Center for Future Intelligent Chips (Chip-X), Nanjing University, China; Suzhou Laboratory, China; National Laboratory of Solid-State Microstructures, School of Electronic Science and Engineering and Collaborative Innovation Center of Advanced Microstructures, Nanjing University, China; The Interdisciplinary Research Center for Future Intelligent Chips (Chip-X), Nanjing University, China; National Laboratory of Solid-State Microstructures, School of Electronic Science and Engineering and Collaborative Innovation Center of Advanced Microstructures, Nanjing University, China; National Laboratory of Solid-State Microstructures, School of Electronic Science and Engineering and Collaborative Innovation Center of Advanced Microstructures, Nanjing University, China; Suzhou Laboratory, China; The Interdisciplinary Research Center for Future Intelligent Chips (Chip-X), Nanjing University, China; School of Integrated Circuits, Nanjing University, China

## Abstract

This Perspective aims to provide a concise survey of current progress and outlook future directions in high-performance transistors and integrated circuits (ICs) based on 2D semiconductors.

Two-dimensional (2D) semiconductors, particularly transition metal dichalcogenides, exhibit high mobility within atomic-scale thickness, immunity to short-channel effects, and back-end-of-the-line (BEOL) compatibility with complementary metal-oxide-semiconductor (CMOS) technology. These attributes have sparked substantial interest in extending the semiconductor roadmap beyond silicon. Major companies including TSMC, Intel, Samsung and IMEC have allocated substantial resources to research into, and development of, 2D-semiconductor technology for advanced technology nodes. The identification of the present development stage and the future target is vital to expedite the transition of 2D semiconductor technology from laboratory to production line. This perspective aims to provide a concise survey of the current progress and future direction of high-performance field-effect transistors (FETs) and integrated circuits (ICs) based on 2D semiconductors.

The International Roadmap for Devices and Systems (IRDS) projects the introduction of 2D semiconductors as channel materials in 0.7 nm nodes in 2034, in a highly scaled three-dimensional (3D) device architecture (Fig. 1a). The basic building block comprises a vertical complementary field-effect transistor (CFET) with gate-all-around (GAA) geometry (Fig. 1b), featuring a gate length of 12 nm and contacted gate pitch (CGP) of 38 nm [1]. Here, CGP denotes the minimal separation between neighboring device gates. Prototype devices, such as multi-bridge channel (MBC) FETs [2], CFETs utilizing 2D semiconductors [3], and MoS_2_ back-gate FETs with a contact pitch as small as 42 nm [4], have been reported. However, their fabrication processes present challenges for scalable and CMOS-compatible production, and their device performance falls short of the IRDS requirements due to unresolved issues in metal contact, dielectric integration, doping, spacer design, etc.

**Figure 1.**
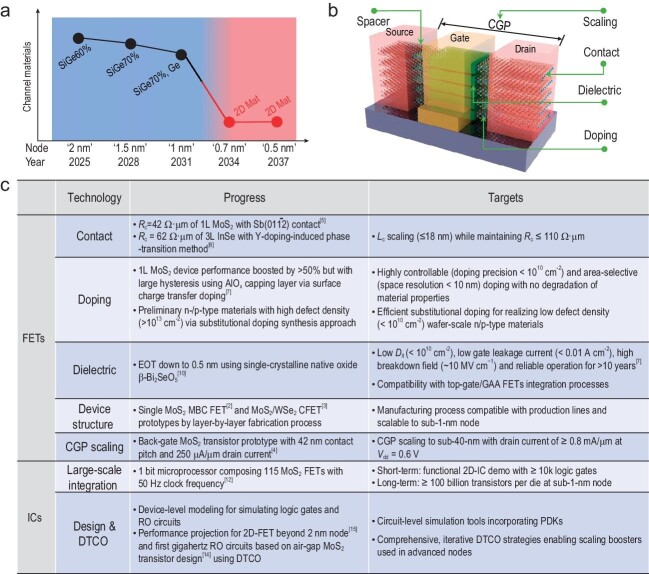
(a) Evolution roadmap of channel materials based on IRDS 2022. (b) Schematic representation of an ideal nanosheet FET highlighting key technologies. (c) Significant advancements and challenges in 2D semiconductor devices and integrated circuits.

Contact resistance (*R*_c_) determines the on-state current of transistors, particularly for short-channel devices. Various methods, such as semimetal contacts [5], 2D van der Waals contacts and Y-doping-induced phase-transition contact [6], have recently been proposed to notably reduce *R*_c_ of 2D semiconductors. Particularly, the semimetal Sb (01$\bar{1}$2) contact achieved a remarkably low *R*_c_ of 42 Ω·μm in monolayer MoS_2_ through orbital hybridization [5]. This value surpasses that of chemically bonded contact in bulk semiconductors and is within a factor of ∼2 from the quantum limit at a carrier density of $3.0 \times {10}^{13}$ cm^−2^. The improved contact results in a current density of 1.23 mA/μm under 1 V source-drain bias. Despite achieving ultralow *R*_c_, several significant challenges for future IC application remain unresolved. A crucial consideration is the contact length (*L*_c_), the overlap between metal electrode and channel, and determining the injection efficiency of charge carriers. Often, low *R*_c_ values are achieved by utilizing *L*_c_ on the micrometer scale. However, in advanced technology nodes, IRDS specifications require *L*_c_ to be sub-20 nm. Although edge contacts with *L*_c_ as short as 1 nm are feasible, their corresponding *R*_c_ still exceeds 10 kΩ·μm. Additionally, conventional *R*_c_ measurements employ a back-gate device structure to establish highly doped contact. Such a device structure, however, introduces parasitic capacitance arising from the overlap between gate and source/drain and will limit the high-frequency performance in circuits. Hence, it is imperative to incorporate spacer design into the structure of 2D semiconductor devices for effectively mitigating parasitic effects, specifically the undesired capacitance and resistance that arise from the interactions between the gate, substrate and source-drain.

In silicon CMOS, the contact and spacer regions undergo heavy doping with a concentration ranging from 10^21^ to 10^22^ cm^−3^ to reduce access resistance while keeping parasitic capacitance low, thereby reducing the resistive-capacitive (RC) delay of the transistor [1]. Consequently, it is imperative to develop controlled and reliable doping techniques for constructing transistors and ICs using 2D semiconductors. Currently, the most promising doping methods [7] include substitutional doping and surface charge-transfer doping. Substitutional doping involves the addition of external donor or acceptor elements during material synthesis, which has the potential to produce p- and n-type materials in batch fabrication. However, the reduced screening in 2D semiconductors leads to much larger dopant ionization energy, thus much lower doping efficiency than bulk semiconductors. Surface charge-transfer doping offers a gentle approach that preserves the structure of 2D materials. CMOS-compatible dopants mainly consist of non-stoichiometric oxides and nitrides, such as SiO_x_, AlO_x_, MoO_x_ and SiN_x_. By employing photolithography, the spatially selective doping of 2D materials becomes achievable, and beneficial for realizing the ideal integrated device structure in Fig. 1b. Yet, these non-stoichiometric oxides might introduce additional long-range optical phonon scattering, and achieving precise control of the doping level remains challenging. Although the recently reported remote modulation doping method addresses this concern to some degree, it appears incompatible for device scaling [8]. Reliable and effective doping methods for 2D semiconductors are still to be developed.

The integration of ultrathin dielectrics in 2D semiconductor devices has experienced remarkable advancements in recent years. From the utilization of organic molecules as an interface buffer layer [9] to the adoption of self-oxidized Bi_2_OSe_5_ [10], the equivalent oxide thickness (EOT) has already met IRDS requirements for a sub-1 nm node. However, the interface trap density (*D*_it_) at the dielectric–2D interface still exceeds that of the Si–SiO_2_ interface (<10^10^ cm^−2^) by ∼2 orders of magnitude. This discrepancy primarily stems from lattice defects and interface impurities, potentially leading to high power dissipation and reliability issues. It is worth noting that the utilization of *in-situ* oxidation to generate a high-κ oxide has the potential to produce an atomically sharp interface [11], akin to Si–SiO_2_. However, devices reported in existing experiments exhibit elevated subthreshold swing (SS) values exceeding 100 mV/dec. The precise control of oxidation conditions, while simultaneously avoiding adverse effects on the underlying 2D channel, continues to pose challenges. In addition, investigations into the reliability of gate dielectrics, a critical factor for industry, remain relatively limited. Besides single-device studies, conducting statistical analyses of yield and uniformity through measurements on multi-batch wafer-level arrays is also essential.

Although significant progress has been made in the performance enhancement of 2D transistors, the integration density of 2D ICs remains low. For example, a 1-bit microprocessor utilizing MoS_2_ transistors was successfully demonstrated [12], featuring 115 transistors in an area of 0.6 mm². In comparison, state-of-the-art CMOS-based CPUs possess over 10 billion transistors in ∼100 mm^2^. This substantial gap is attributed to the immaturity of highly crystalline 2D semiconductor materials, the manufacturing process of device integration and circuit design environment (Fig. 1c). These areas all necessitate continuous research and development by academia and industry.

For materials, the production of large (up to 12-inch) wafer-scale single-crystalline 2D semiconductor materials still poses a significant challenge, although proof-of-concept demonstration of 2-inch MoS_2_, MoSe_2_ and WS_2_ single crystals has been reported [13]. Key considerations include meticulous substrate engineering, growth thermodynamics and kinetics, equipment design and process control. To achieve success in this field, it is crucial to engage in iterative processes throughout the growth process and prioritize the development of specialized equipment, distinct from the commonly employed tube furnaces found in academic laboratories. Besides, a viable wafer-scale, non-destructive transfer technology is required to relocate 2D materials from growth substrate to integration platform in an ultra-clean environment. Ideally, this technique should take place in a vacuum.

To facilitate the lab-to-fab transition of 2D semiconductors, there is an urgent need for the advancement of standard device-integration processes tailored for large-scale manufacturing. Besides the aforementioned issues, processes related to the interconnection/stacking of 2D semiconductor transistors and circuits must also be developed, including etching, vias, thin film deposition, atomic layer deposition/etching, chemical mechanical polishing, passivation and encapsulation. It is important to highlight that all of these processes must meet stringent requirements, including compatibility with CMOS technology, as well as the prevention of structural damage and interface contamination in 2D semiconductors. Adopting a more forward-looking viewpoint, it is possible that growth, fabrication, integration equipment and systems operating within a high-vacuum environment might ultimately evolve into the optimal solution for the future manufacturing of 2D semiconductor ICs.

Circuit design plays a crucial role in Very Large-Scale Integration (VLSI). While some efforts have been dedicated to device-level modeling, there remains a lack of comprehensive circuit-level simulation tools that incorporate process design kits (PDKs) to assess circuit performance. Additionally, although many studies have provided statistical information regarding yield and variability [5], it is important to establish clear definitions of yield criteria and acceptable variability windows. Furthermore, addressing the challenges faced by 2D semiconductors in VLSI requires more than just design-related solutions; it necessitates the adoption of a design technology co-optimization (DTCO) methodology. Recently, we adopted DTCO to report the first gigahertz ring oscillator (RO) circuits based on air-gap MoS_2_ transistor design [14]. Looking forward, a process-aware DTCO approach is expected to yield a >40% improvement in the operating frequency performance of a 15-stage ring oscillator with a fan-out of 3 at 0.7 V supply in 2D-FET technology, as compared to silicon, at the IMEC 2 nm node [15].
